# Experimental Investigation of Uranium and Iron Condensation from High-Temperature Plasma Conditions

**DOI:** 10.1021/acsomega.5c13649

**Published:** 2026-03-12

**Authors:** Emily N. Weerakkody, Zurong Dai, Kate E. Rodriguez, Mark A. Burton, Timothy P. Rose, Batikan Koroglu, Enrica Balboni

**Affiliations:** Physical and Life Sciences Division, 4578 Lawrence Livermore National Laboratory, Livermore, California 94550, United States

## Abstract

We used a plasma flow reactor (PFR) to generate synthetic fallout nanoparticles from vapor-phase condensation of two different input concentrations of uranium and iron analytes (U/Fe = 1:1 and 1:2). Synthetic fallout from complex chemical matrices (e.g., mixtures of U and Fe) has not been generated in this setup before and allows for the observation of relative condensation and fractionation of nuclear debris. Experiments were conducted under two different temperature histories with variations in particle flow patterns along the PFR. Transmission electron microscopy (TEM) observation and analysis of the nanoparticles revealed variations in speciation of uranium oxides (UO_2_ and α-UO_3_) depending on the competition between flow mixing and oxygen sequestration by iron. A ternary metal oxide, UFeO_4_, was observed in addition to iron oxides (e.g., FeO and Fe_3_O_4_), which suggests that fallout models should account for chemical speciation of ternary metal oxides (i.e., UFeO_4_) and their relative condensation behaviors in addition to those of singular metal oxides (e.g., FeO and UO_2_). X-ray Energy Dispersive Spectroscopy (EDS) elemental maps showed that some particles had Fe-rich cores surrounded by U-rich regions. This suggests that either condensed U oxides coagulate onto molten Fe oxides or that the increase in iron analyte concentration might drive the system toward a higher degree of supersaturation, leading to earlier formation of iron oxide particles and providing an energetically favored pathway for nucleation of uranium oxides around iron oxide particles.

## Graphical abstract

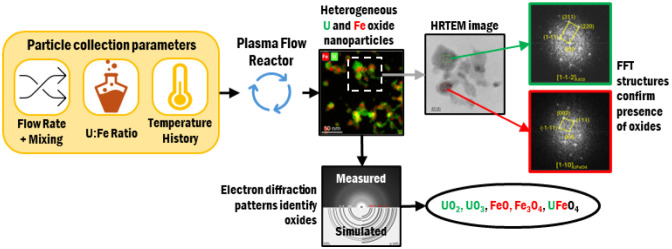

## 1 Introduction

The nuclear fireball is an extreme environment in which complex chemistry occurs between a number of constituents under high-temperature and turbulent flow conditions.^1^ Chemical constituents include species that originate from the nuclear device itself and the surrounding environment. Many factors, such as the height of burst, initial temperature/cooling rate, and yield of a nuclear detonation, affect the size, dispersion, and chemical composition of fallout debris particles, including their radionuclide content.^2–7^ These factors directly influence the fate and transport of fallout, as well as its impacts on exposed populations.^4^

Analyses of debris particles collected from nuclear tests have revealed that fallout forms through various physical pathways, including condensation from the vapor phase and coagulation on the surface of molten entrained material.^8,9^ Although nuclear debris forms in a high-energy environment, it has been recognized for decades that its chemical and isotopic composition rarely, if ever, represents a fully homogenized mixture of fireball products. Instead, as the debris cools and forms, chemical fractionation processes lead to the separation of constituents. The many chemical species (atoms, molecules, ions, etc.) and their recombined forms (e.g., oxides) present in the postdetonation fireball environment have distinct properties (including vapor pressure and volatility) that influence condensation of chemically complex debris particles.^6,7,9,10^ The chemical fractionation processes in the nuclear fireball are not constant from event to event, and their effects on debris composition are difficult to predictively model.^6,11,12^

There have been many efforts to characterize fallout particles.^13–19^ Studies of postdetonation nuclear debris have uncovered chemical and structural inhomogeneities, revealing complex formation mechanisms. One study showed that the oxidation states of U, Fe, and Pu in test debris varied depending on the amount of Fe present and cooling rates.^13,14^ Researchers have also observed variable distributions of radionuclides within different compositional components of macroscopic debris particles.^15–17^ Weisz et al. modeled U and Si deposition to determine how glassy fallout may have rapidly evolved in the aftermath of a nuclear fireball and incorporated other species, such as Ca and Al, during this process.^18^ Genda et al. observed phase separation in Fe- and Si-containing fallout collected from historic near-surface tests, resulting in complex amoeboid microstructures in the Fe-rich rims of mm-scale fallout samples, indicating that vapor-phase Fe-rich material deposited on molten silicates as products cooled from the postdetonation fireball.^19^ Flow conditions in the fireball also play a critical role in determining the mechanisms of particle coagulation, which influence fallout formation. Laminar flow promotes shear flow collisions, while turbulent flow introduces accelerative effects, with particle size, inertia, settling velocity, and phase (solid or liquid) further shaping the nature and structure of the resulting fallout debris.^2^ Evidence of mixing and flow conditions, such as compositional flow textures, coagulation, and vesiculation, can be preserved in debris.^10,19^ These complex findings underscore the need to study the pathways involved in fallout formation.

Laboratory testbeds have been developed to emulate a postdetonation fireball environment, lending insight into the complexities of nuclear debris formation. A common testbed is the laser-produced plasma (LPP),^20,21^ which has been used to probe materials of interest to nuclear fireballs, such as U^22–29^ and Pu,^30,31^ as well as alloys like U_3_Si_2_ ^8,32,33^ to establish chemical pathways for these systems containing multiple constituents in relevant conditions. However, due to the short-lived nature of LPPs (μs time scale), these systems cannot adequately replicate the time scales of the nuclear fireball nor do they allow for variation of flow conditions. The plasma flow reactor (PFR) is another testbed that provides steady-state monitoring of chemical pathways from rapidly cooling plasmas but at rates (ms time scales)^34–38^ that are more relevant to nuclear fireball dynamics. In the PFR platform, gaseous species can be probed in situ spectroscopically, and condensed nanoparticles can be collected and analyzed using techniques such as transmission electron microscopy (TEM) and scanning electron microscopy (SEM). Thus far, PFR studies have looked at the condensation of singular metallic nitrate analytes (e.g., U, Al, Ce, and Fe) and variations in oxygen content, flow rate, and temperature histories to determine the effects on speciation and sizing of the resulting particulates.^34,35,39^ Synthetic fallout from more complex chemical matrices (e.g., mixtures of U and Fe), which is important for understanding the role of relative condensation behaviors of metal oxides in chemical fractionation of nuclear debris and establishing the relevant chemical pathways in multiphysics fallout models, has not been generated before using the PFR setup. The condensation behavior of such mixtures is not well understood, and this is the first attempt to study relevant phenomena using this apparatus.

The present study investigated the condensation behaviors of U and Fe mixtures in the PFR to improve our understanding of their physical and chemical interactions during particle formation from a cooling plasma. Iron is commonly present in nuclear debris^13,14,40,41^ due to its abundance in industrial materials and its use in rigging historic tests. Like U, Fe is a redox-sensitive element, and its presence could potentially alter the speciation of U products formed in a postdetonation environment, particularly in tandem with the extreme flow conditions in such high-energy environments. We conducted this study using mixtures of 1:1 and 1:2 U and Fe by atom ratios and analyzed the resulting particulates using TEM-based techniques to investigate how species availability, including the amount of Fe and oxygen present, as well as different flow conditions (varied flow rates and temperature histories), affected their formation, sizing, speciation, and distribution at relevant time scales. This is the first attempt to study condensation of mixed actinide and metal analytes using this methodology.

Our findings suggest that the presence of Fe influences speciation of U oxides by providing competition for oxygen present in the system and alters the morphology and structure of the nanoparticles generated. Metal oxides (UO_2_, UO_3_, FeO, and Fe_3_O_4_) form in varying proportions and incorporate together, depending on the flow conditions of the system. Furthermore, mixed U–Fe oxides (e.g., UFeO_4_) can form in these extreme environments. Current multiphysics fallout models only account for monometallic oxides, but results from this study indicate that mixed oxide formation should be incorporated into said models.

## 2 Experimental Methods

### 2.1 Plasma Flow Reactor and Test Conditions

The plasma flow reactor (PFR) at Lawrence Livermore National Laboratory is a unique experimental apparatus that features an inductively coupled plasma (ICP) that allows for the introduction of aqueous analytes at varied concentrations. This system includes changeable outermost flow rates to create different temperature histories (Figure 1). A peristaltic pump delivers aqueous analyte into the nebulizer spray chamber, which aerosolizes the analyte. The innermost and outermost flows are introduced at the RF (radio frequency) coil, which generates the inductively coupled plasma. The innermost flow injects the aerosolized analyte into the plasma. The outermost argon flow directs the plasma down the ∼1 m long reactor tube. A ring flow injector couples the ICP torch to the reactor tube about 4 cm downstream of the plasma and injects additional argon to increase the cooling rate along the PFR, as described in previous publications.^34–39,42^

**Figure 1:**
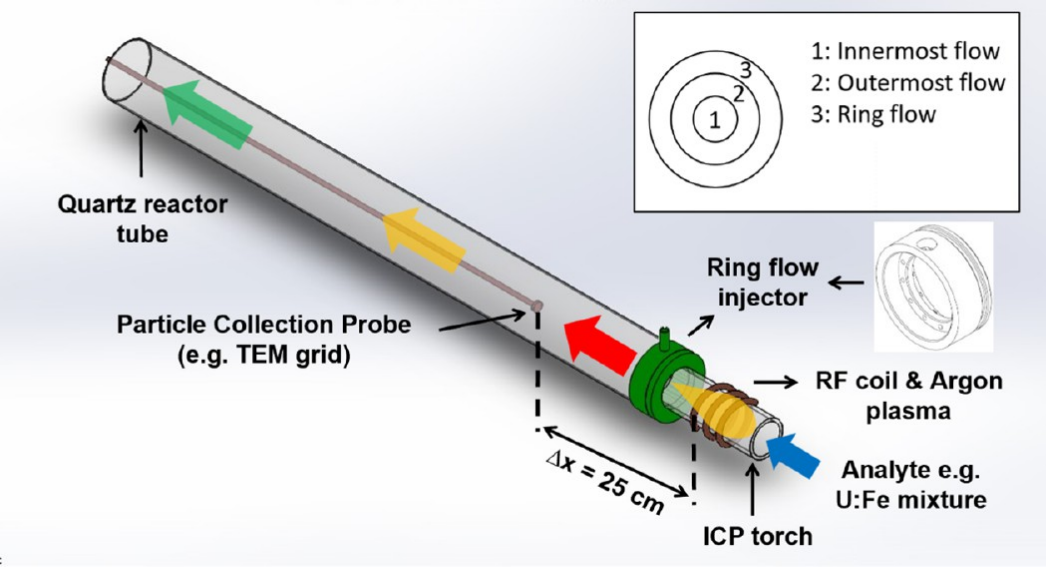
Plasma flow reactor schematic adapted from Rodriguez et al.^35^ The PFR consists of an inductively coupled plasma torch through which argon gas and nebulized analytes flow. The plasma can be probed by in situ methods like optical spectroscopy and ex situ by characterizing collected particles. Figure reproduced with permission from the ACS, 2026.

In the PFR, the analyte is atomized by a ∼5000 K plasma^37,39^ and condenses into particulates, which further aggregate as the constituents cool. Prior to the experiments, the temperature at different distances from the RF coil was measured using K-type thermocouples centered within the reactor tube and water as the analyte for the two flow conditions. Prior experiments demonstrated that the injected analyte does not affect the temperature of the system at a given downstream (>20 cm) location.^35^ As shown in Figure 2, an outermost flow rate of 12.1 L/min leads to a higher starting plasma temperature compared to a flow rate of 14.4 L/min.^39^

**Figure 2:**
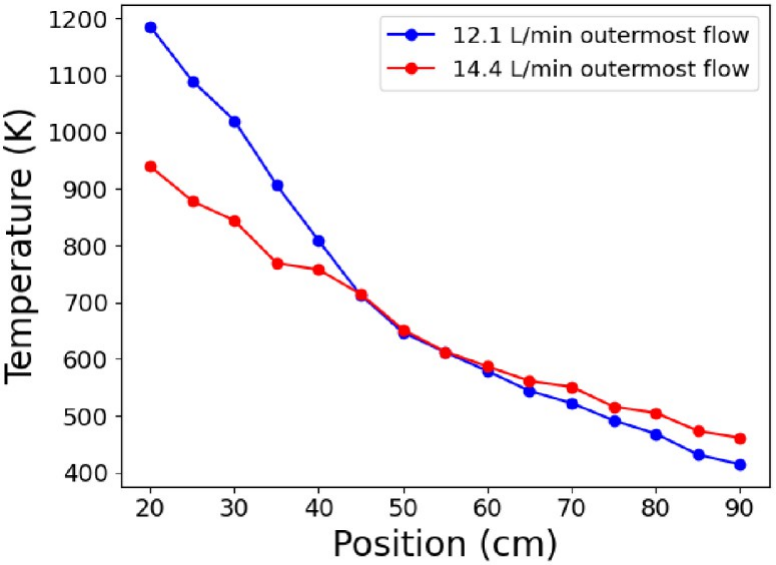
K-type thermocouples were used to obtain temperatures for the different outermost flow conditions tested. Temperatures of downstream locations from 20 to 90 cm from the RF coil are shown. The blue trace corresponds to the 12.1 L/min outermost flow rate. The red trace corresponds to the 14.4 L/min outermost flow rate. Prior work contains expanded temperature information from other flow conditions and further upstream.^35,39^ Figure reproduced from Weerakkody et al.^42^ with permission from the ACS, 2026.

The five test cases conducted in this work are summarized in Table 1. Aqueous solutions containing U nitrate hexahydrate (SPI Supplies, CAS #13520-83-7, 98–100%) and Fe nitrate nonahydrate (Sigma-Aldrich, CAS #7782-61-8, 98–100%) were prepared such that the ratios of U:Fe atoms were 1:1 and 1:2 with distilled water. The solutions contained 0.125 g/mL U and 0.1 g/mL Fe for the 1:1 case (Cases #1, #2, and #2B) and 0.125 g/mL U and 0.2 g/mL Fe for the 1:2 cases (Cases #3, #4). The concentration of U was chosen to be comparable to previous studies.^39,42^ Atom ratios in Table 1 were determined from analyte and gas inputs to the flow reactor. With the exception of Ar, which was introduced by gas flow, all other elements originated from the U and Fe nitrate precursors and water used to prepare the solutions. Outermost flow varied between 12.1 (Cases #1, #3) and 14.4 L/min (Cases #2, #2B, #4). In all cases, the ring flow was set to 0 L/min (no change in cooling rate), and the innermost flow was set to 1 L/min. Case #2B had an extended collection time in order to gather more material—and therefore stronger electron diffraction patterns—to aid with the identification of the species present in the nanoparticulate.

**Table 1:** Sample Collection Conditions for This Study^a^

			**Atomic ratios**						
**Case**	**Outermost flow (L/min)**	**Collection time (min)**	**U**	**Fe**	**O**	**Ar**	**N**	**H**	**Total concentration of nitrate analytes (g/mL)**
#1	12.1	3	1	1	257	9 × 10^4^	5	479	0.225
#2	14.4	3	1	1	257	10 × 10^4^	5	479	0.225
#2B	14.4	13	1	1	257	10 × 10^4^	5	479	0.225
#3	12.1	3	1	2	275	9 × 10^4^	8	497	0.325
#4	14.4	3	1	2	275	10 × 10^4^	8	497	0.325

a U:Fe atomic ratio and outermost flow were varied. The atomic ratios represented below are calculated from analyte and gas inputs to the flow reactor. Ar comes from the induced gas flow, and all other elements come from the analyte solutions.

### 2.2 Particle Collection and TEM Analysis

To collect particles along the PFR for ex situ analysis, a particle collection probe is inserted from the end of the reactor tube opposite the ICP torch and centered within the reactor tube. The distance between the probe and the RF coil can be varied; however, for the tests in this study, particles were collected at a distance of 25 cm away from the ICP torch on standard silicon nitride grids (Ted Pella P/N: 21502-10) mounted normal to the flow in an aluminum TEM grid holder (Ted Pella: #15469). The temperature at this position was ∼1100 K for the 12.1 L/min outermost flow and ∼880 K for the 14.4 L/min outermost flow conditions. This position was chosen to maximize the amount of particulate collected as well as to be directly comparable to prior experiments.^37,39,42^ In this study, the particle collection probe was inserted into the PFR at the specified distance, the ICP was turned on, and then the analyte was introduced for particle collection for the designated amount of time (13 min for Case #2B and 3 min for all other cases). Once collection was completed, the ICP was turned off, the probe was removed, and the TEM grid was prepared for ex situ analysis.

TEM was used to characterize the particles obtained to evaluate their composition, distribution, sizing, and crystallinity. An FEI Titan 80-300 S/TEM with a Super-X G-2 X-ray energy dispersive system was utilized to this end. Here, bright-field and dark-field TEM imaging, high-resolution transmission electron microscopy (HRTEM), selected area electron diffraction patterns (SADP), high-angle annular dark field (HAADF) scanning transmission electron microscopy (STEM), and energy dispersive X-ray spectroscopy (EDS) elemental mapping were applied. ImageJ was used to process TEM/HAADF-STEM images for size distribution, as discussed in previous publications.^35,39^ Briefly, images taken at the same magnification and in different locations on the TEM grid were processed, and a Python script was used to create particle size distribution plots for comparison between different test cases.

## 3 Results and Discussion

### 3.1 Particle Morphology and Size Distribution

#### 3.1.1 Particle Morphology

Representative brightfield TEM images of particles synthesized from Cases #1–#4 are shown in Figure 3. The top row shows particles generated from injecting the 1:1 atom ratio mixture of U and Fe analyte into the PFR (Cases #1 and #2), and the bottom row shows particles generated from the 1:2 atom ratio mixture (Cases #3 and #4). The columns correspond to outermost flow rates of 12.1 and 14.4 L/min.

**Figure 3:**
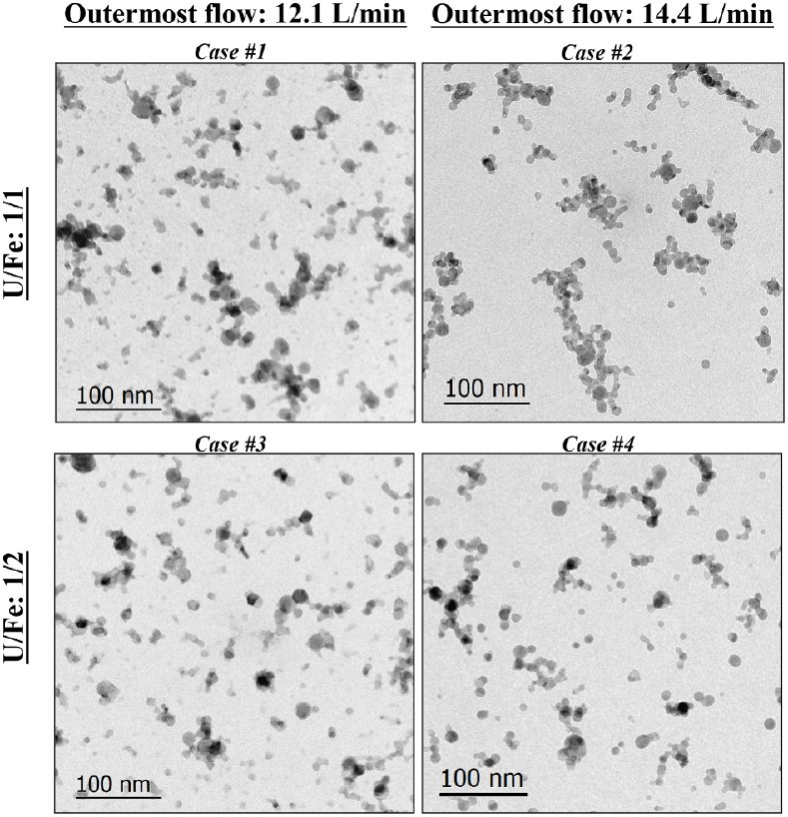
Brightfield TEM images from Cases #1–#4 showing particle morphology. The top row is from the 1:1 U:Fe analyte, and the bottom row is from the 1:2 U:Fe analyte. The columns correspond to the different outermost flow rates.

For all cases, particles largely present a spherical or quasi-spherical shape with varying sizes, distributing as isolated particles or aggregates to varying degrees. Cases #1 and #3 feature a mixture of single particles and shorter chained aggregates, whereas particles in Cases #2 and #4 appear mostly spherical, with relatively more abundant and larger aggregates/chains (100s nm) and fewer small, isolated particles (<5 nm). Cases #1 and #3 have the lower outermost flow rate (12.1 L/min). The low outermost flow rate may potentially reduce both the collision rate and the resulting particle aggregation.

The particle morphology observed in all cases is generally consistent with that reported by Rodriguez et al.^39^ for U oxides, as well as that reported by Koroglu et al.^37^ for iron (although lower-resolution SEM images were reported in that study). All cases in this study exhibit a higher particle number density and a qualitatively greater degree of particle aggregation compared to U-only experiments. We hypothesize that this increase may be due to the presence of Fe in the system, possibly as a result of the added Fe increasing the nucleation rate and favoring heterogeneous nucleation of particles.^43^

Figure 4 shows a comparison between the shorter (3 min) and extended collection durations for the U-only (10 min) and 1:1 U:Fe (13 min) cases with the outermost flow rate of 14.4 L/min. Previous studies with U as the sole analyte^42^ demonstrated that increasing particle collection time increases the amount of particles deposited on the substrate, while their speciation remains unchanged.

**Figure 4:**
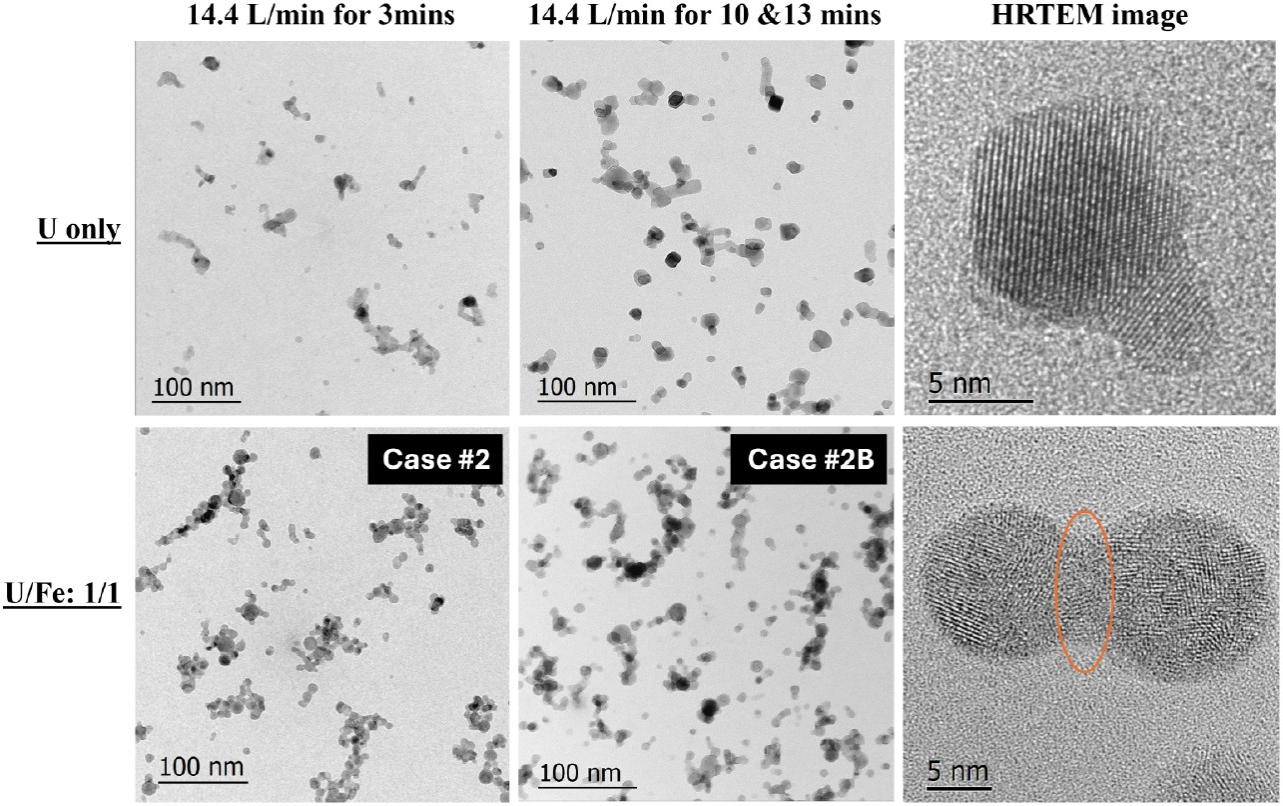
Brightfield TEM and HRTEM images showing a morphological comparison of particles formed between U as the only analyte (3 and 10 min)^39,42^ and the 1:1 U:Fe analyte mixture at 14.4 L/min outermost flow rate for different collection durations (3 min—Case #2 and 13 min—Case #2B). The orange oval indicates potential melting between particles.

From these images, it appears that increased collection time leads to larger particles and that this effect seems more pronounced in the U:Fe mixtures. Particles from the U-only case, particularly for the extended duration of collection, mostly have a euhedral shape and are more faceted, whereas individual particles from the 1:1 U:Fe case (Case #2B) are spherical. The HRTEM images of individual particles reveal that the U-only case produced larger single crystals (more homogeneous nucleation), but when Fe was introduced, particles exhibited nanodomain structures, which might indicate more heterogeneous nucleation. The spherical shape of the individual particles in the U/Fe mixture cases implies that these particles may have formed from molten droplets or underwent a melting process. The HRTEM image on the bottom right of Figure 4 indicates two spherical particles which were fused together. The nanodomain structure could form through a precipitation process from the molten droplets. The addition of Fe, therefore, seems to have a significant impact on the nucleation, morphology, and structure of the particles.

#### 3.1.2 Particle Size Distribution

HAADF-STEM images were used to determine the particle size distribution for each of the 3 min duration test cases (Cases #1–4). For each case, images were carefully selected to ensure that the resolution was sufficient to accurately capture the observed particle size. Images with too low magnification were avoided, as they could result in inaccurate measurements and discretization errors due to pixel limitations. To account for the varying number of images collected for each test case, results were normalized such that each case represented an equal effective area, following a similar approach to Rodriguez et al.^35^ Particle areas were obtained using ImageJ software, and particle diameters were calculated as if the particles were spherical.

The normalized particle size distributions, as shown in Figure 5, indicate that the cases with lower outermost flow rates (Cases #1 and #3, see left column) produced more smaller particles overall. Higher outermost flow is expected to lead to better mixing conditions, as has been shown in comparable experimental setups,^38,44^ and results in an increase in particle aggregation and size. In contrast, altering the atomic ratio of U to Fe (from 1:1 to 1:2, see the right column of Figure 5) did not significantly affect the particle sizes, suggesting that particle size is relatively insensitive to the amount of Fe added within this range.

**Figure 5:**
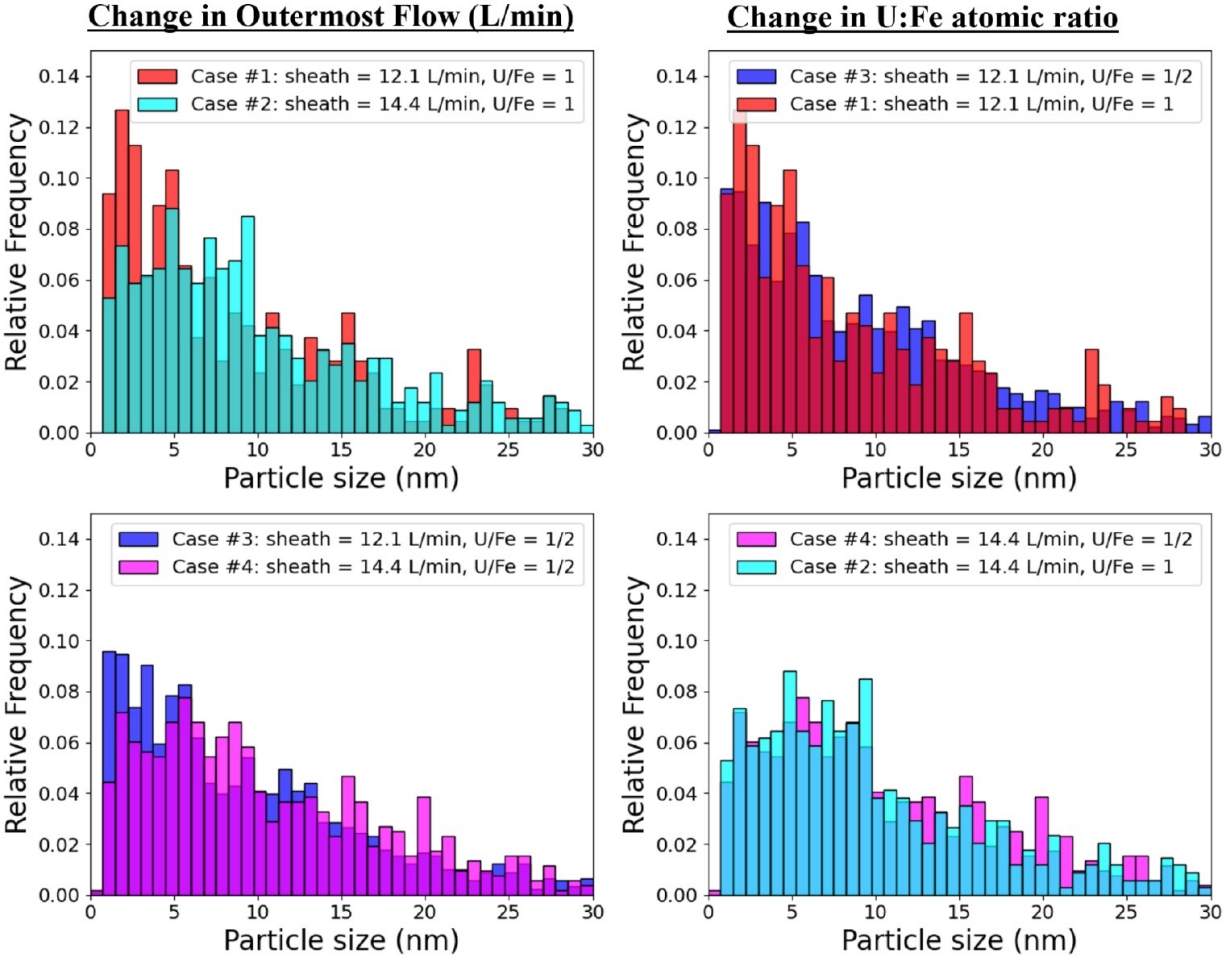
Comparison of particle size distribution formed under each test case. The left column shows change in outermost flow for the different U:Fe ratio conditions present in injected analytes, and the right column pertains to change in U:Fe for the different outermost flows.

Key particle sizing parameters for the different test cases are summarized in Table 2. Mean and median particle areas and diameters are presented, as well as the percent area covered by particulate matter for the images acquired. Standard errors are reported for some metrics. Results from previous work on U speciation under the same conditions are also included for comparison.^39^ Insufficient information was available on Fe oxide particle sizing in prior work^37^ to include in Table 2; however, general trends are discussed. It is important to note that the particle sizing method used here cannot distinguish between aggregated particles and single large particles, so the true average particle sizes may be somewhat lower than those reported.

**Table 2:** Particle Sizing Summary of Cases from This Study and a Comparable Study^39^ with U as the Only Analyte

**Sample:** outermost flow/ratio U:Fe	**Average particle area (nm^2^)**	**Median particle area (nm^2^)**	**Average particle diameter (nm)**	**Median particle diameter (nm)**	**% area coverage in images**	**Number of particles**	**Scaled number of particles**
**Case #1:** 12.1/1:1	126.0 ± 14.0	34.1	9.5 ± 0.5	6.6	8.85	293	913
**Case #2:** 14.4/1:1	181.7 ± 19.5	56.8	11.3 ± 0.5	8.5	7.78	473	657
**Case #3:** 12.1/1:2	153.1 ± 8.1	49.3	10.5 ± 0.3	7.9	14.25	1276	1624
**Case #4:** 14.4/1:2	171.8 ± 13.1	65.3	11.6 ± 0.3	9.1	6.90	715	715
*12.1*/**U only** ^39^	47	9.5	5.7	3.5	-	695	-
*14.4*/**U** **only** ^39^	107	36.4	9.0	6.8	-	1189	-

The data show that the presence of Fe leads to a significant increase in particle size compared to that in U-only cases. Additionally, higher outermost flow rates, which correspond to lower initial temperatures and greater mixing, result in the formation of larger particles. However, among the cases with the higher outermost flow of 14.4 L/min (Cases #2 and #4), increasing the Fe content does not produce a meaningful difference in average particle area or size distribution.

Previous work^37^ has shown that PFR-synthesized Fe oxide nanoparticles exhibit a bimodal size distribution, with most particles around 25 nm and some as large as 150 nm, while U oxide particles tend to be smaller and more uniform in size. The average particle sizes reported in this study for U/Fe mixtures fall between those previously published for pure U^39^ and pure Fe^37^ oxide nanoparticles.

The number of particles produced per effective area varied between the different test conditions. Notably, Case #3 (12.1 L/min outermost flow, 1:2 U/Fe ratio) exhibited the highest number of particles and the greatest percent area coverage. In contrast, Case #4 showed a lower percent area coverage, suggesting that less material was collected under these conditions. These differences in area coverage between cases may result from radial variations in flow associated with changes in the outermost flow rate. Both higher-flow cases (#2 and #4) demonstrated reduced area coverage, which could be attributed to increased mixing that may promote the formation of larger particles less likely to deposit on the centered TEM substrate. This interpretation aligns with previous findings that higher outermost flow enhances mixing,^39^ leading to more frequent particle collisions and the formation of larger aggregates, as well as a different spatial distribution of particles within the reactor and plasma.^42^

### 3.2 Fe and U Elemental Distribution and Particle Speciation

#### 3.2.1 Fe and U Elemental Distribution in Particle Aggregates

X-ray EDS elemental mapping was used to obtain the spatial distribution of U- and Fe-based constituents across each test case. The resultant X-ray EDS elemental maps are shown in Figures 6 and 7 for Cases #1–#4 and Figure 8 for Case #2B. The mosaic maps of Fe–K and U–M in Figures 6 and 8 reveal that the distribution of uranium (U) and iron (Fe) elements varies from particle to particle—some particles are U-rich (green), and others are Fe-rich (red).

**Figure 6:**
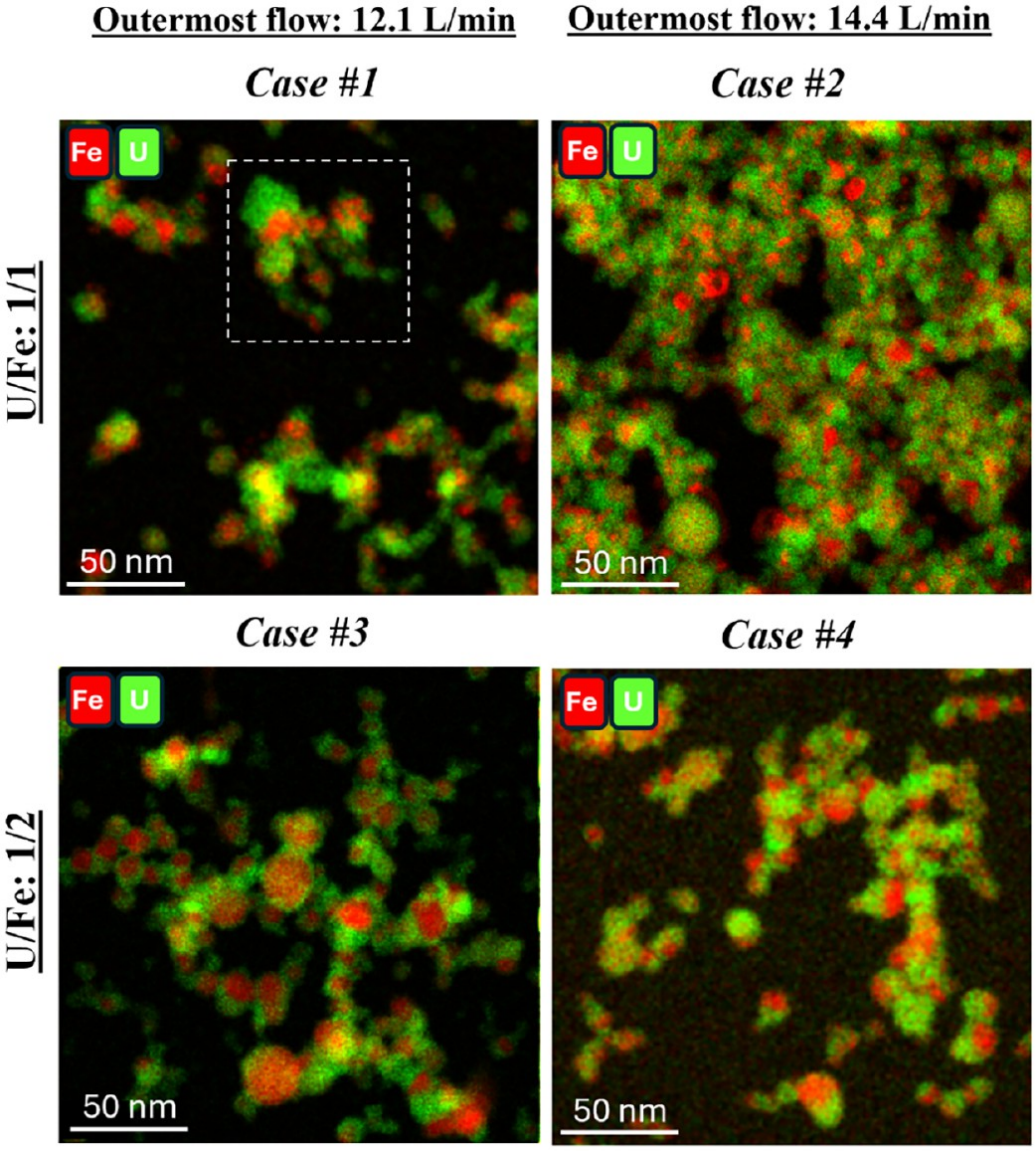
Mosaic X-ray EDS maps of elemental Fe (red) and U (green) are shown for the different test cases with a 3 min collection time at similar magnification (Cases #1–4). Particle sizes for the faster outermost flow (14.4 L/min) appear smaller than those of the slower outermost flow case (12.1 L/min). The 1:2 U:Fe cases appear much more Fe-rich and well-distributed. The white box in Case #1 denotes a region that will be discussed in further detail in a later figure.

**Figure 7:**
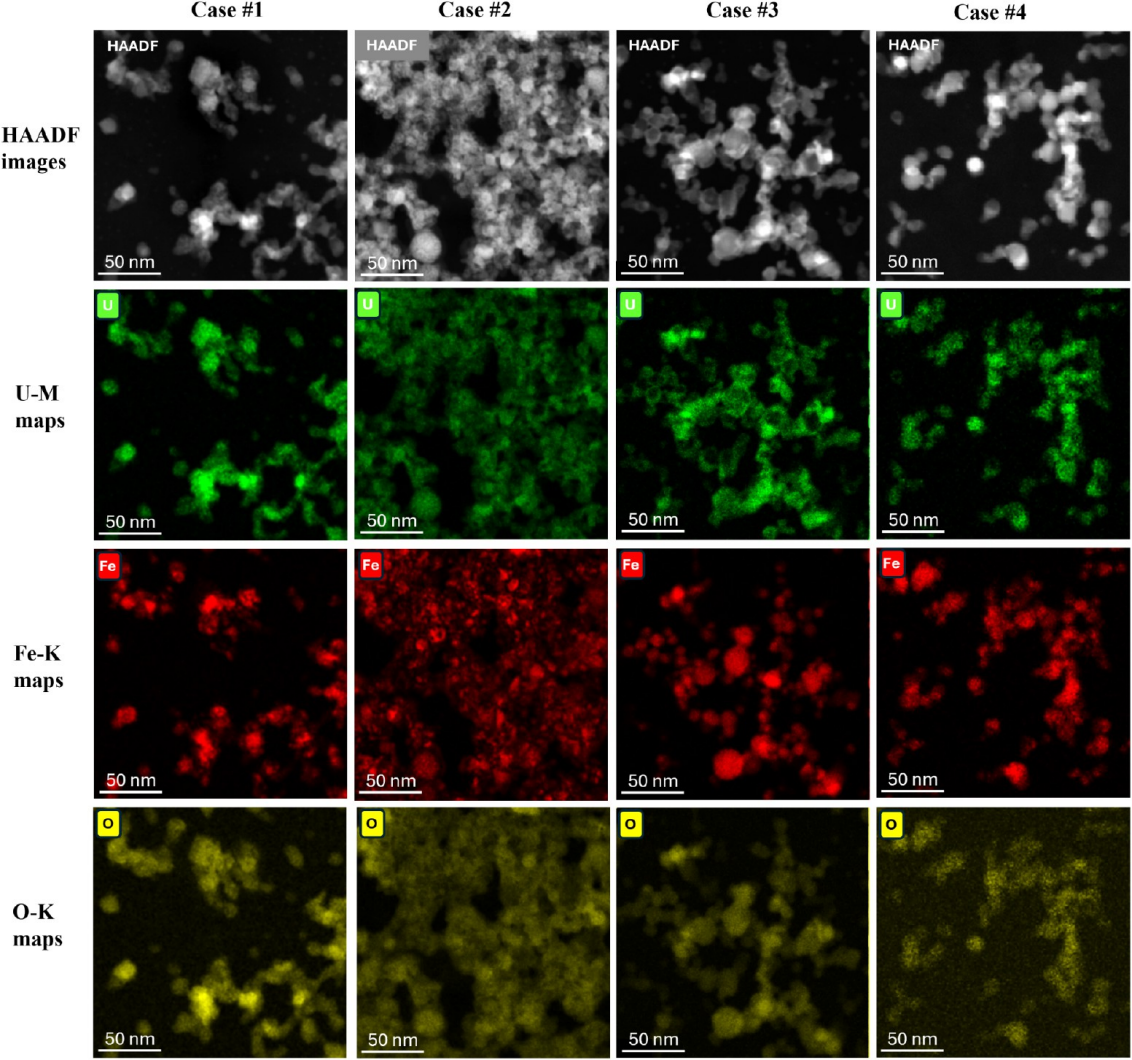
HAADF STEM images and X-ray EDS elemental maps of U–M, Fe–K, and O–K from Cases #1–4. These images show that the U-rich regions appear to encase the Fe-rich regions.

**Figure 8:**
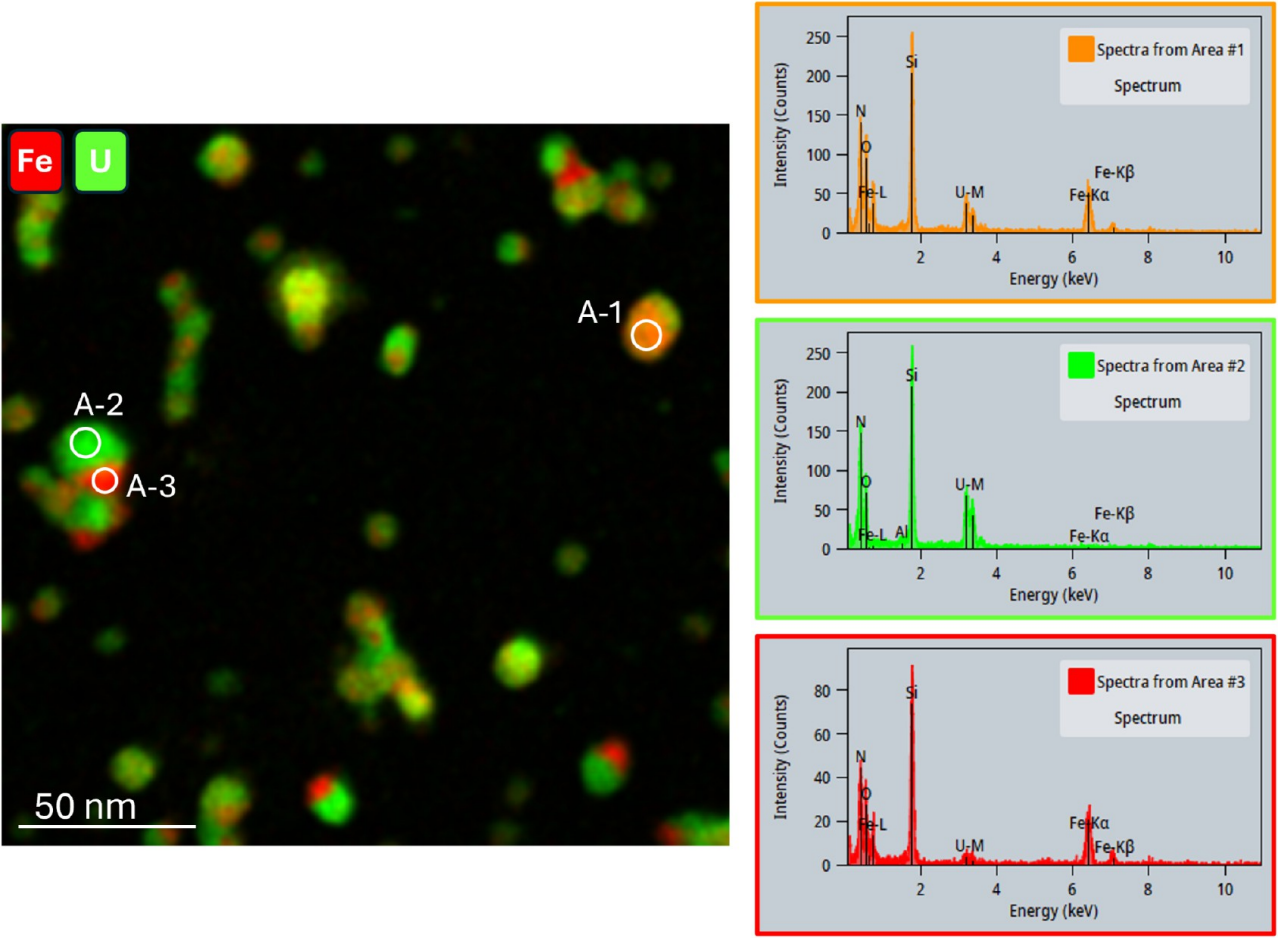
Mosaic X-ray map of elemental Fe (red) and U (green) from Case #2B (14.4 L/min, 13 min collection). EDS spectra extracted from the representative areas (A-1, A-2, A-3) indicate variations in the relative amounts of Fe and U present from particle to particle in a single sample—even within a single aggregate. The N and Si shown in the spectra originate from the silicon nitride supporting film.

For the 1:1 U:Fe mixture, Fe-rich particles appear as smaller isolated aggregates at the lower outermost flow rate (Case #1). In contrast, the cases with higher Fe content (Cases #3 and #4, U/Fe: 1/2) display well-defined Fe-rich regions—there appears to be an increase in the degree of supersaturation of iron with an increase in iron content. Higher outermost flow rates (14.4 L/min) result in more homogeneous distributions of Fe-rich and U-rich regions (Cases #2 and #4), consistent with previous findings that increased flow rates enhance constituent mixing.^38,39,44^

The separate elemental maps of U, Fe, and O in Figure 7 allow us to better understand how these elements are distributed within the particles and aggregates. For example, these maps confirm that particles formed in all test cases are metal oxides, and Fe-rich regions appear more localized, cohesive, and spherical than the U-rich regions, potentially indicating melt-phase formation of Fe oxides. The images also suggest that U-rich regions tend to encase Fe-rich particles. Such behavior is reminiscent of nuclear debris evolution, where cooling vapor condenses onto preexisting particles or solid particles formed at higher temperatures (U-oxides) may coagulate onto or around liquid droplets of lower melting point (Fe-oxide).^19,43^ The coagulation of U oxides onto the surface of an Fe oxide droplet could help explain why the average particle sizes in this study fall between those formed for U and Fe as sole analytes under the same flow conditions.

#### 3.2.2 Particle Speciation: Electron Diffraction Analysis

To further understand speciation and phase constitution of the particles formed under various test conditions, X-ray EDS spectra were extracted from three representative areas of the elemental map for Case #2B, as shown in Figure 8. The qualitative analysis indicates that the U/Fe ratio dramatically varies between the different areas selected. Area-1 (orange) contains comparable amounts of U and Fe; Area-2 (green) is rich in uranium but contains almost no detectable iron; and Area-3 (red) is rich in iron with extremely low uranium content. These findings suggest that the synthesized particles may contain phases of U-oxide, Fe-oxide, and potentially U–Fe oxide.

SADPs were obtained to determine the crystal structure and composition of the nanoparticles collected for all test cases, with results for Cases #1–#4 illustrated in Figure 9 and Case #2B in Figure 10. Tabulated electron diffraction measurements and phase identifications for Case 1 are provided in Table 3 as an example. Case #2B was collected under the same conditions as Case #2 but for an extended collection time to clarify species signatures that were only weakly observed in Case #2. A prior study on U oxide formation indicated that the duration of collection in the PFR does not affect particle speciation but does increase the amount of particles deposited on the substrate.^42^ The greater particle deposition on the TEM substrate enhances the signal-to-noise ratio in SADP, leading to stronger reflection signals and more reliable phase identification (Figure 9). Please note that particulate from Fe as the sole analyte was synthesized at 14.4 L/min and collected at 16 cm from the ICP torch (unlike 25 cm in all other cases).^34^ The temperature at this location was ∼1100 K (similar to the 12.1 L/min, 25 cm in our work) in that study due to a water-cooled copper shield encasing the PFR, which is no longer in use.

**Figure 9:**
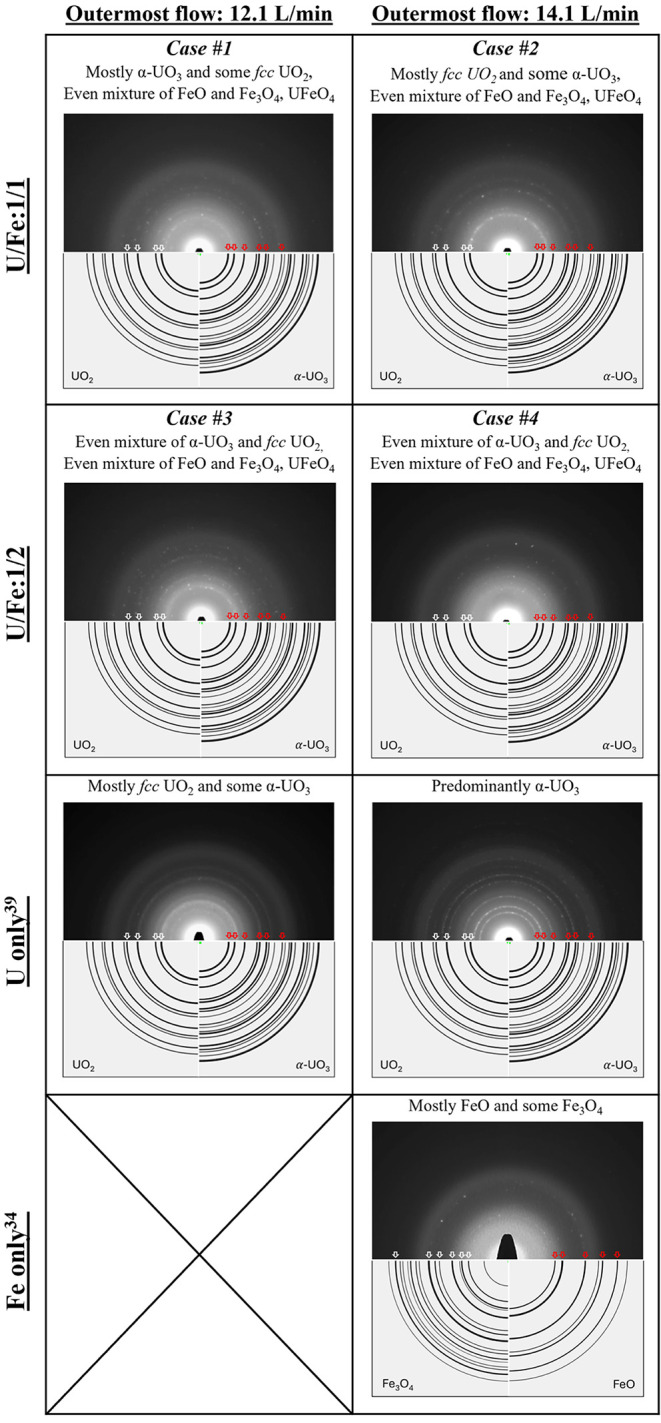
Selected-area electron diffraction patterns for each of the U:Fe test cases with 3 min collection time, as well as patterns for U and Fe as singular analytes from prior studies.^39,42^ Particulate from Fe as the sole analyte^34^ was synthesized at 14.4 L/min and collected at 16 cm from the ICP torch (unlike 25 cm in all other cases). Diffraction patterns for U only are reproduced from Rodriguez et al.^39^ with permission from ACS 2026.

**Figure 10:**
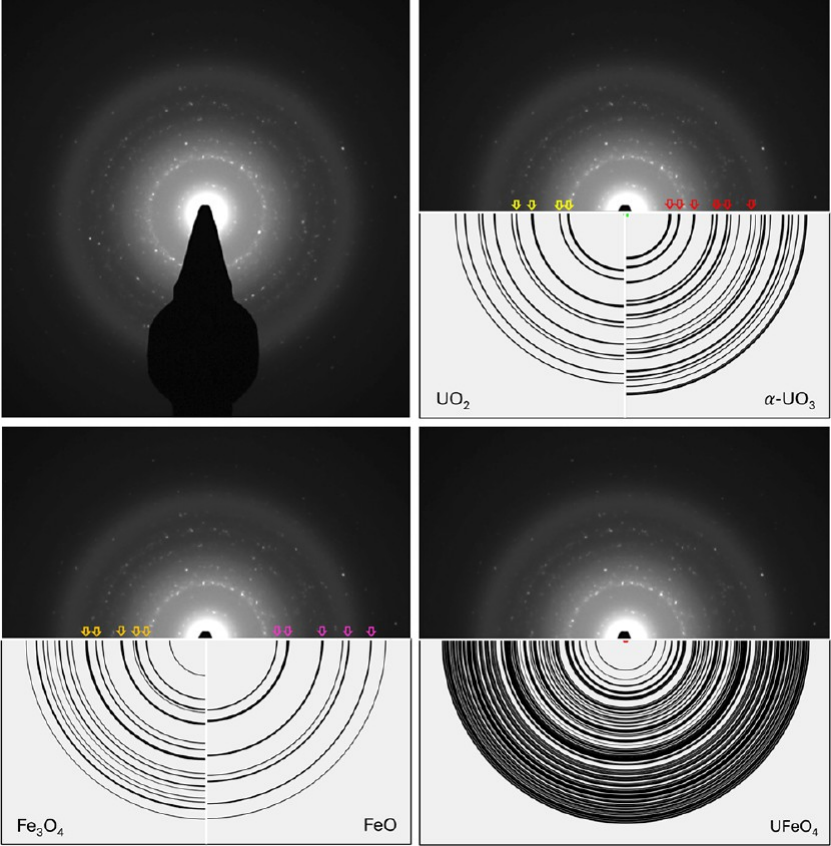
Selected-area electron diffraction pattern corresponding to Case #2B, 14.4 L/min and 1:1 U:Fe with 13 min collection time, including reference diffraction patterns for UO_2_ (yellow), α-UO_3_ (red), Fe_3_O_4_ (orange), FeO (purple), and UFeO_4_.

**Table 3:** Electron Diffraction Measurement and Phase Identification for Case #1^a^

		**UFeO_4_ (A)**			**UO_2_ (B)**			**α-UO**_3_**** **(C)**			**FeO (D)**			**Fe**_**3**_ **O**_**4**_ **(E)**		
**Experimental measurement**		PDF#_04-013-3762			PDF#_00-067-0020			PDF#_04-005-9770			PDF#_01-089-7100			PDF#_01-080-6402		
*d* (Å)	Phase	*d* (Å)	Int (%)	*hkl*	*d* (Å)	Int (%)	*hkl*	*d* (Å)	Int (%)	*hkl*	*d* (Å)	Int (%)	*hkl*	*d* (Å)	Int (%)	*hkl*
5.99	A	5.9685	7	020												
														4.8454	10	111
		4.5235	6	110												
								4.1700	54	001						
3.84	A	3.8817	35	021												
3.45	C							3.4520	100	100						
3.38	A	3.3870	49	111												
3.16	B				3.1583	100	111									
3.07	A	3.0858	100	130												
2.94	A, E	2.9843	4	040										2.9672	30	220
2.78	B				2.7352	34	200									
2.65	A, C	2.6416	4	131				2.6591	77	011						
2.54	A, E	2.5770	23	041										2.5304	100	311
		2.5550	15	002												
2.47	A, D	2.4440	10	200							2.4895	67	111	2.4227	7	222
2.35	A	2.3488	2	022												
2.22	A, D	2.1452	3	150							2.1560	100	200			
2.07	A, C, E	2.0682	12	221				2.0850	8	002				2.0981	21	400
1.98	A, C	1.9895	4	060				1.9930	28	110						
		1.9780	10	151												
		1.9680	26	132												
1.93	A, B	1.9408	3	042	1.9341	56	220									
1.86	A	1.8908	3	240												
1.83	C							1.7982	27	111						
1.77	A, C	1.7733	21	241				1.7847	32	012						
		1.7661	14	202												
1.71	C, E							1.7260	12	200				1.7131	9	422
1.65	A, B	1.6429	3	152	1.6494	64	311									
		1.6379	3	023												
1.62	A, E	1.6101	3	170										1.6151	27	511
1.59	A, C	1.5941	8	113				1.5948	14	021						
1.56	A, B	1.5697	3	113	1.5792	19	222									
		1.5429	3	260												
		1.5394	8	311												
		1.5368	8	171												
1.51	A, D	1.5078	5	330							1.5245	47	220			
1.48	A, E	1.4793	6	043										1.4836	36	440

a All crystal structure data were obtained from the powder diffraction file (PDF) in the International Centre for Diffraction Data (ICDD) database.^45^

For indexing and interpreting SADPs, all commonly known phases of U and Fe intermetallic compounds and oxides were considered, including UFe_2_, UFe_6_, U-oxides, Fe-oxides, UFe_2_O_6_, U_3_FeO_10_, and UFeO_4_. As a result, *fcc* UO_2_, α-UO_3_, FeO, Fe_3_O_4_, and UFeO_4_ were identified in each sample, with variations in relative amounts due to changes in flow conditions and input atomic ratios.

The U oxide particles identified were predominantly crystalline, whereas Fe oxides exhibited relatively poor crystallinity that varied between samples, as shown in the high-resolution TEM images in Figure 4, where the spherical individual particles with nanodomains and amorphous surfaces may be indicative of molten droplet formation. The phase characterization is summarized in Table 4. Although extracting quantitative information from SADP is challenging, it is possible to gain insight into the relative amounts of different species and their degrees of crystallinity.

**Table 4:** Detailed Summary of Speciation under Different Test Cases Obtained from the SADP Technique^a^

	**Outermost flow**	
**Species**	**12.1 L/min** **(∼1100 K)**	**14.4 L/min** **(∼880 K)**
**U/Fe:** 1/1	**Case #1:**	**Case #2 and 2B:**
	• Mostly *α-UO*_*3*_ and some *fcc UO*_*2*_	• Mostly *fcc UO*_*2*_ and some *α-UO*_*3*_
	• Even mixture of *FeO* and *Fe*_*3*_ *O*_*4*_	• Even mixture of *FeO* and *Fe*_*3*_ *O*_*4*_
	• *UFeO*_*4*_	• *UFeO*_*4*_
**U/Fe: 1/2 (increased number density)**	**Case #3:**	**Case #4:**
	• Even mixture of *α-UO*_*3*_ and *fcc UO*_*2*_	• Even mixture of *α-UO*_*3*_ and *fcc UO*_*2*_
	• Even mixture of *FeO* and *Fe*_*3*_ *O*_*4*_	• Even mixture of *FeO* and *Fe*_*3*_ *O*_4_
	• *UFeO*_*4*_	• *UFeO*_*4*_
**U** ^39^	• Mostly *fcc UO*_*2*_ and some *α-UO*_*3*_	• Predominantly *α-UO*_*3*_
**Fe** ^34^		• Mostly *FeO* and some *Fe*_*3*_ *O*_*4*_ (*∼1100 K*)

a Results from Rodriguez et al. for cases containing only U and results from Koroglu et al. from experiments with Fe as the sole analyte^34^ are included for comparison.^39^ Temperatures at the collection point for each experiment are noted for each condition.

#### 3.2.3 Effects Due to Addition of Fe

Under 12.1 L/min outermost flow with U as the sole analyte,^39^ mostly UO_2_ forms with some α-UO_3_. When Fe is added under the same flow conditions and U content such that U:Fe = 1:1 (Case #1), α-UO_3_ becomes the dominant U oxide observed. Although more oxygen is available in the system due to the Fe nitrate analyte addition, the additional Fe competes with U for this oxygen, resulting in the formation of FeO, Fe_3_O_4_, and UFeO_4_. When more Fe is added under the same flow conditions (U:Fe = 1:2, Case #3), a more even mixture of U oxides is observed along with Fe oxides and UFeO_4,_ perhaps due to increased collision frequency and competition for oxygen. It is important to consider that other oxygen-containing species can form in the flow reactor (e.g., H_2_O, O_2_, etc.), which would remain in the gas phase and flow into the exhaust of the PFR.^46^

Previous work has shown that UFeO_4_ can form from reactions between U oxides and Fe oxides at elevated temperatures (e.g., 1673 K) under reducing and oxidizing conditions, including reactions of UO_3_ + FeO.^47^ While these conditions^47^ are not fully analogous to the present study, it is plausible that UFeO_4_ is forming upstream of the collection from high-temperature reactions of U and Fe oxides. Fe_2_O_3_ was not observed in these experiments and in previous experiments with Fe as the sole analyte.^34^ Fe_2_O_3_ may have been absent due to high temperatures at the collection point (too high for Fe_2_O_3_ condensation) and its formation requiring a much higher oxygen concentration (∼60 at% O) than what was available in these experimental conditions (∼0.3 at% O).^48^ The presence of uranium may also have prevented the higher oxidation of Fe. Phase change temperatures for species relevant to this study are shown in Table 5.

**Table 5:** Phase Change Temperatures of Species Relevant to This Study

**Species**	**Condensation (K)**	**Decomposition (K)**	**Boiling/Sublimation (K)**
U^49,50^	∼1400	-	∼4400
UO_2_ ^51^	∼3120	∼2673	∼4100
UO_3_ ^52^	∼900–1100	∼1150–1250	∼1800–2000
Fe^53,54^	∼1811	-	∼3134
FeO^55^	∼1357	∼1640	∼3414
Fe_2_O_3_ ^56^	∼800–900	∼1565	∼3020–3150
Fe_3_O_4_ ^57^	∼1000–1100	∼1870	∼2980–3100
UFeO_4_ ^58^	-	∼1113	-

#### 3.2.4 Effects Due to Increased Ar Flow Rate (Decreased Temperature/Increased Mixing)

Increasing the outermost flow from 12.1 L/min (Case #1) to 14.4 L/min (Case #2) shifts the dominant U oxide from α-UO_3_ to UO_2_. The shift of the dominant U oxide from α-UO_3_ to UO_2_ between Case #1 and Case #2 is likely due to several factors, including changes in temperature history, reduced oxygen availability due to the higher argon flow rate, and oxygen sequestration by Fe. Although the temperature at the collection point (25 cm downstream of the RF coil) decreases by approximately 200 K between the two flow rates, previous studies have shown that the cooling effect is even more pronounced upstream,^37^ which can slow the conversion of UO_2_ to α-UO_3_. In previous experiments with U as the sole analyte, higher outermost flow rates promoted the formation of α-UO_3_, attributed to enhanced mixing and increased U oxidation.^39,42^ In the present study, elemental mapping of U and Fe confirms that a higher outermost flow improves reactant mixing (Figure 6). However, the presence of Fe in the system leads to oxygen sequestration, favoring the formation of UO_2_ over that of α-UO_3_ under otherwise similar conditions. This suggests that oxygen availability,^59^ influenced by both increased argon flow and Fe oxide formation, is the primary factor driving the observed shift in U oxide speciation, with temperature playing a secondary role.

#### 3.2.5 Combined Effects

A comparison of Cases #1 and #3 suggests that the added Fe may sequester more oxygen, resulting in a decreased proportion of α-UO_3_. However, the data from Case #4 complicate this interpretation, as the relative amounts of UO_2_ and α-UO_3_ are nearly equivalent. Previous studies with U-only systems have shown that increased mixing tends to promote the formation of α-UO_3_.^39^ In contrast, under similar flow conditions, the introduction of Fe to the starting solution increases the formation of UO_2_, as observed when comparing Case #1 to Case #3. The combined effect of increased mixing and added Fe creates conditions that are more favorable to UO_2_ formation, as seen when comparing Case #1 vs Case #4. In U-only experiments, an abundance of oxygen and enhanced mixing may facilitate the formation of higher U oxides, such as α-UO_3_, due to the absence of competition from other metals. In this study, both U and Fe are simultaneously forming oxides, and increased mixing from Case #1 to Case #2 likely promotes the formation of Fe oxides and U–Fe oxides, potentially at the expense of higher U-oxides. These observations, along with findings from previous studies, emphasize the sensitivity of U-oxide formation to local redox conditions.^34,35,37–39,42^

#### 3.2.6 Fast Fourier Transform Analysis of Particles

To further elucidate the distributions of U and Fe oxides within particles, TEM samples from Case #1 (1:1 U/Fe, 12.1 L/min flow, and 3 min collection) were investigated at increased resolution. High-resolution transmission electron microscopy (HRTEM) lattice images were captured under different defocusing conditions to image a target particle that overlapped with another particle. The fast Fourier transform (FFT) of the HRTEM image was then used for the phase identification of oxide particles. Figure 11 shows this analysis applied to the region indicated by the white box in Figure 6. X-ray EDS maps suggest that U-oxide regions may be encasing Fe-oxide regions. HRTEM imaging and FFT analysis revealed particles corresponding to *fcc* UO_2_ (Figure 11g and h) and UFeO_4_ (Figure 11i and j), respectively. Notably, regions containing only Fe oxide species (FeO and Fe_3_O_4_) were not identified using HRTEM imaging with FFT analysis, indicating that Fe oxides are well mixed with or obscured by other species, because their main *d*-spacings overlap with those of other phases, particularly UFeO_4_ (Table 3). For this reason, α-UO_3_ is also difficult to identify using this technique. The identification of a distinct UFeO_4_ region in this aggregate is particularly significant for fallout modeling applications, which typically consider singular metallic oxides as condensation products rather than oxides containing multiple metals.

**Figure 11:**
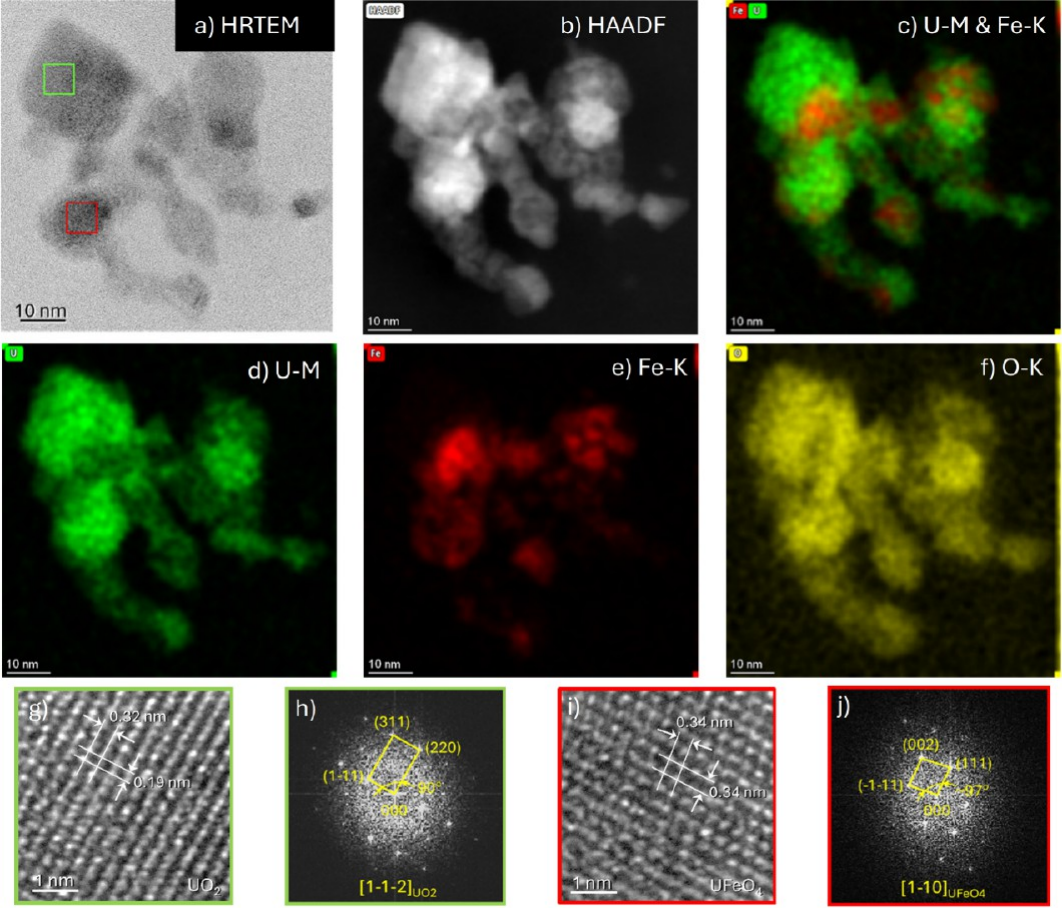
High-resolution TEM analysis and X-ray EDS mapping on the region indicated by the white box in Figure 6 for Case #1 (12.1 L/min, 3 min collection): a) HRTEM image with FFT areas highlighted; b) HAADF STEM image; c) mosaic image of U–M and Fe–K; d) X-ray EDS maps of U–M, e) Fe–K, and f) O–K; g) HRTEM; h) FFT of the region corresponding to the green box overlaid on the HRTEM image (a) matching fcc UO_2_; i) HRTEM image; and j) FFT of the region corresponding to the red box overlaid on the HRTEM image (a) matching UFeO_4_.

Identification of the *fcc* UO_2_ region in Figure 11 further demonstrates that *fcc* UO_2_ tends to coagulate on the exterior of Fe-rich regions, despite the higher condensation temperatures of U oxides compared to Fe oxides (Table 5) Although FeO and Fe_3_O_4_ have lower condensation temperatures and are less refractory than UO_2_, experimental observations indicate that UO_2_ coagulates on the outside of the Fe-rich regions, possibly in conjunction with α-UO_3_, which has a lower condensation temperature than the Fe oxides. An increase in Fe concentration may also lead to supersaturation—iron particles may form earlier, and nucleation of U oxides around Fe oxide particles may be more energetically favorable.

## 4 Conclusions

In summary, U oxide phase formation is sensitive to local redox conditions, which are created by the interplay of flow mixing, temperature history, and oxygen availability in the PFR. The presence of Fe influences U oxide speciation by sequestering oxygen, resulting in the preferred formation of *fcc* UO_2_ relative to higher oxide forms (e.g., α-UO_3_). Increased argon flow rate seems to enhance mixing and promote further reaction and aggregation of vapor-phase species as they condense, increasing both particle size and promoting a more even distribution of U and Fe oxide species. SADP and FFT analyses confirmed the presence of crystalline U oxides (UO_2_ and α-UO_3_), poorly crystalline Fe oxides (FeO and Fe_3_O_4_), which may indicate melt phase formation, and UFeO_4_. The proportions and incorporation of these species vary with the flow conditions induced in the system. As in previous studies with the PFR, Fe_2_O_3_ was not detected under these conditions, possibly due to the high collection temperature and relatively oxygen-poor environment. X-ray EDS mapping at various magnifications revealed that condensed U oxides encase the Fe oxides. Distinct regions of UO_2_ and UFeO_4_ in nanoparticles were confirmed using FFT, although isolated Fe oxide regions could not be identified using this technique, indicating that they are incorporated within or obscured by other species, which is supported by the X-ray EDS findings. Coagulation of condensed U oxides onto liquid Fe oxides may explain both the observed chemical distribution of Fe and U in the particles and the increase in particle size with the addition of Fe—the presence of Fe may reduce the mobility of U species in fallout. Interactions between U and Fe oxide species as they condense clearly influence their distribution and ultimately particle sizing, which plays a significant role in fallout dispersal. The presence of UFeO_4_ suggests that fallout models should account for the formation of multimetal species in addition to monometallic oxides (e.g., FeO and UO_2_) under these extreme conditions. Perhaps in the study of other mixtures, we may uncover more multimetal species formation in these environments.

There are many avenues for future work. The study of other mixtures of interest to fallout formation is already underway (i.e., U/Cs and U/Al/Si). Material substrates could be introduced downstream of the plasma to better simulate the nucleation of particulates onto molten substances, as observed in a study of historic debris. Other temperature histories could be probed by the addition of ring flow (cooling) and the use of a tube furnace to slow the cooling rate as particles travel down the PFR to determine the effects of the cooling rate on the condensation of mixed species. Nanoparticles can be collected at points further downstream in the flow reactor to determine variations as the species continue to cool. Spectroscopic diagnostics could be used to confirm the fate of O-containing gaseous species in the PFR.

## References

[1] Effects of Nuclear Weapons, 3rd ed., Glasstone, S. ; Dolan, P. J. ; United States Department of Defense, 1977.

[2] McGuffin D. L., Lucas D. D., Balboni E., Nasstrom J. S., Lundquist K. A., Knight K. B. (2024). Predictive Modeling of Atmospheric
Nuclear Fallout
Microphysics. Sci. Total Environ..

[3] Freiling E. C., Kay M. A. (1966). Radionuclide Fractionation
in Air-Burst Debris. Nature.

[4] Freiling, E. C. ; Crocker, G. R. ; Adams, C. E. . Radioactive Fallout From Nuclear Weapons Tests; EPA: Germantown, MD, 1965.

[5] Freiling E. C. (1961). Radionuclide
Fractionation in Bomb Debris. Science.

[6] Miller, C. F. . A Theory of Formation of Fallout from Land-Surface Nuclear Detonations and Decay of the Fission Products; Naval Radiological Defense Laboratory: San Francisco, CA, 1960.

[7] Edvarson K., Löw K., Sisefsky J. (1959). Fractionation Phenomena in Nuclear
Weapons Debris. Nature.

[8] Weisz D. G., Crowhurst J. C., Finko M. S., Rose T. P., Koroglu B., Trappitsch R., Radousky H. B., Siekhaus W. J., Armstrong M. R., Isselhardt B. H., Azer M., Curreli D. (2018). Effects of Plume Hydrodynamics
and Oxidation on the Composition of a Condensing Laser-Induced Plasma. J. Phys. Chem. A.

[9] Adams C. E., Farlow N. H., Schell W. R. (1960). The Compositions,
Structures and
Origins of Radioactive Fall-out Particles. Geochim.
Cosmochim. Acta.

[10] Weisz D. G., Jacobsen B., Marks N. E., Knight K. B., Isselhardt B. H., Matzel J. E., Weber P. K., Prussin S. G., Hutcheon I. D. (2017). Deposition
of Vaporized Species onto Glassy Fallout from a Near-Surface Nuclear
Test. Geochim. Cosmochim. Acta.

[11] Moody, K. J. ; Hutcheon, I. D. ; Grant, P. M. Nuclear Forensics Applications; Taylor and Francis: Boca Raton, FL, 2005.

[12] Dardenne, Y. ; Parker, W. ; Knight, K. B. Chemical Fractionation Is Not a Constant: Revisiting Bomb Vapor Chemistry USDOE National Nuclear Security Administration (NNSA) 2020

[13] Pacold J.
I., Lukens W. W., Booth C. H., Shuh D. K., Knight K. B., Eppich G. R., Holliday K. S. (2016). Chemical Speciation of U, Fe, and
Pu in Melt Glass from Nuclear Weapons Testing. J. Appl. Phys..

[14] Giuli, G. ; Pratesi, G. ; Eeckhout, S. G. ; Koeberl, C. ; Paris, E. Iron reduction in silicate glass produced during the 1945 nuclear test at the Trinity site (Alamogordo, New Mexico, USA); Geological Society of America: (Alamogordo, New Mexico, USA), 2010.

[15] Bellucci J. J., Simonetti A., Koeman E. C., Wallace C., Burns P. C. (2014). A Detailed
Geochemical Investigation of Post-Nuclear Detonation Trinitite Glass
at High Spatial Resolution: Delineating Anthropogenic vs. Natural Components. Chem. Geol..

[16] Bellucci J. J., Simonetti A. (2012). Nuclear Forensics:
Searching for Nuclear Device Debris
in Trinitite-Hosted Inclusions. J. Radioanal.
Nucl. Chem..

[17] Belloni F., Himbert J., Marzocchi O., Romanello V. (2011). Investigating
Incorporation and Distribution of Radionuclides in Trinitite. J. Environ. Radioact..

[18] Weisz D. G., Jacobsen B., Marks N. E., Knight K. B., Isselhardt B. H., Matzel J. E. (2018). Diffusive Mass Transport
in Agglomerated Glassy Fallout
from a Near-Surface Nuclear Test. Geochim. Cosmochim.
Acta.

[19] Genda T., Knight K., Dai Z. R., Balboni E., Goldblum B. L., Hosemann P. (2021). Iron-Rich Microstructure Records of High Temperature
Multi-Component Silicate Melt Behavior in Nuclear Fallout. J. Environ. Radioact..

[20] Amoruso S., Bruzzese R., Spinelli N. (2017). Laser-Induced Plasma Emission: From
Atomic to Molecular Spectra Characterization of Laser-Ablation Plasmas. J. Phys. Appl. Phys.

[21] Hahn D. W., Omenetto N. (2012). Laser-Induced Breakdown
Spectroscopy (LIBS), Part II:
Review of Instrumental and Methodological Approaches to Material Analysis
and Applications to Different Fields. Appl.
Spectrosc..

[22] Kautz E. J., Weerakkody E. N., Finko M. S., Curreli D., Koroglu B., Rose T. P., Weisz D. G., Crowhurst J. C., Radousky H. B., De Magistris M., Sinha N., Levin D. A., Dreizin E. L., Phillips M. C., Glumac N. G., Harilal S. S. (2021). Optical
Spectroscopy and Modeling of Uranium Gas-Phase Oxidation: Progress
and Perspectives. Spectrochim. Acta Part B At.
Spectrosc..

[23] Burton M. A., Auner A. W., Crowhurst J. C., Boone P. S., Finney L. A., Weisz D. G., Koroglu B., Jovanovic I., Radousky H. B., Knight K. B. (2022). The Effect of Oxygen
Concentration
on the Speciation of Laser Ablated Uranium. Sci. Rep..

[24] Harilal S. S., Kautz E. J., Bernacki B. E., Phillips M. C., Skrodzki P. J., Burger M., Jovanovic I. (2019). Physical Conditions
for UO Formation
in Laser-Produced Uranium Plumes. Phys. Chem.
Chem. Phys..

[25] Skrodzki P. J., Shah N. P., Taylor N., Hartig K. C., La Haye N. L., Brumfield B. E., Jovanovic I., Phillips M. C., Harilal S. S. (2016). Significance
of Ambient Conditions in Uranium Absorption and Emission Features
of Laser Ablation Plasmas. Spectrochim. Acta
- Part B At. Spectrosc..

[26] Burger M., Finney L. A., Garrett L., Harilal S. S., Hartig K. C., Nees J., Skrodzki P. J., Xiao X., Jovanovic I. (2021). Laser Ablation
Spectrometry for Studies of Uranium Plasmas, Reactor Monitoring, and
Spent Fuel Safety. Spectrochim. Acta Part B
At. Spectrosc..

[27] Finko M. S., Curreli D. (2018). Simulation of Uranium Plasma Plume Dynamics in Atmospheric
Oxygen Produced via Femtosecond Laser Ablation. Phys. Plasmas..

[28] Finko M. S., Curreli D., Weisz D. G., Crowhurst J. C., Rose T. P., Koroglu B., Radousky H. B., Armstrong M. R. (2017). A Model
of Early Formation of Uranium Molecular Oxides in Laser-Ablated Plasmas. J. Phys. D: Appl. Phys..

[29] Campbell K. R., Wozniak N. R., Colgan J. P., Judge E. J., Bare J. E., Kilcrease D. P., Wilkerson M. P., Czerwinski K. R., Clegg S. M. (2017). Phase Discrimination
of Uranium Oxides Using Laser-Induced
Breakdown Spectroscopy. Spectrochim. Acta, Part
B.

[30] Harilal S. S., Murzyn C. M., Kautz E. J., Edwards M. K., Sinkov S. I., Bisson S. E., Mitra S. S., Martin J. B. (2021). Spectral Dynamics
and Gas-Phase Oxidation of Laser-Produced Plutonium Plasmas. J. Anal. At. Spectrom..

[31] Miyabe M., Oba M., Jung K., Iimura H., Akaoka K., Kato M., Otobe H., Khumaeni A., Wakaida I. (2017). Laser Ablation Absorption
Spectroscopy for Isotopic Analysis of Plutonium: Spectroscopic Properties
and Analytical Performance. Spectrochim. Acta
- Part B At. Spectrosc..

[32] Weerakkody E. N., Weisz D. G., Crowhurst J., Koroglu B., Rose T., Radousky H., Stillwell R. L., Jeffries J. R., Glumac N. G. (2020). Time-Resolved
Formation of Uranium and Silicon Oxides Subsequent to the Laser Ablation
of U3Si2. Spectrochim. Acta - Part B At. Spectrosc..

[33] Goodall P., Johnson S. G., Wood E. (1995). Laser Ablation
Inductively Coupled
Plasma Atomic Emission Spectrometry of a Uranium-Zirconium Alloy:
Ablation Properties and Analytical Behavior. Spectrochim. Acta Part B At. Spectrosc..

[34] Koroglu B., Wagnon S., Dai Z., Crowhurst J. C., Armstrong M. R., Weisz D., Mehl M., Zaug J. M., Radousky H. B., Rose T. P. (2018). Gas Phase Chemical
Evolution of Uranium,
Aluminum, and Iron Oxides. Sci. Rep..

[35] Rodriguez K., Koroglu B., Hammons J., Dai Z., Ferrier M. G., Balboni E., Rose T., Knight K. B. (2022). Vapor-Phase
Aggregation
of Cerium Oxide Nanoparticles in a Rapidly Cooling Plasma. ACS Earth Space Chem..

[36] Koroglu B., Mehl M., Armstrong M. R., Crowhurst J. C., Weisz D. G., Zaug J. M., Dai Z., Radousky H. B., Chernov A., Ramon E., Stavrou E., Knight K., Fabris A. L., Cappelli M. A., Rose T. P. (2017). Plasma
Flow Reactor
for Steady State Monitoring of Physical and Chemical Processes at
High Temperatures. Rev. Sci. Instrum..

[37] Koroglu B., Finko M., Saggese C., Wagnon S., Foster S., McGuffin D., Lucas D., Rose T. P., Crowhurst J. C., Weisz D. G., Radousky H. B., Curreli D., Knight K. B. (2022). The Influence
of Cooling Rate on Condensation of Iron, Aluminum, and Uranium Oxide
Nanoparticles. J. Aerosol Sci..

[38] Koroglu B., Dai Z., Finko M., Armstrong M. R., Crowhurst J. C., Curreli D., Weisz D. G., Radousky H. B., Knight K. B., Rose T. P. (2020). Experimental Investigation
of Uranium Volatility during
Vapor Condensation. Anal. Chem..

[39] Rodriguez K., Weerakkody E. N., Dai Z., Knight K. B., Koroglu B., Rose T. P., Balboni E. (2023). The Influence of Temperature History
and Flow Mixing on the Vapor-Phase Speciation of Uranium Oxide Nanoparticles. ACS Earth Space Chem..

[40] Stewart K. (1956). The Condensation
of a Vapour to an Assembly of Droplets and Particles. Trans. Faraday Soc..

[41] Heft, R. E. The Characterization of Radioactive Particles from Nuclear Weapons Tests. In Radionuclides in the Environment; WASHINGTON, 1970, pp. 254–281. DOI: 10.1021/ba-1970-0093.

[42] Weerakkody E. N., Koroglu B., Dai Z., Rodriguez K. E., Balboni E. (2024). Dependence of Uranium Oxide Polymorphism
on Plasma
Synthesis Conditions. J. Phys. Chem. A.

[43] Moresco, P. Description of Nucleation, Growth and Coagulation Processes in the Modeling of Debris Formation after a Nuclear Burst; ORNL 2021. DOI: 10.2172/1760119.

[44] Shin J. W., Miyazoe H., Leparoux M., Siegmann S., Dorier J. L., Hollenstein C. (2006). The Influence
of Process Parameters on Precursor Evaporation
for Alumina Nanopowder Synthesis in an Inductively Coupled Rf Thermal
Plasma. Plasma Sources Sci. Technol..

[45] Kabekkodu S. N., Dosen A., Blanton T. N. (2024). PDF-5+: A Comprehensive Powder Diffraction
File^TM^ for Materials Characterization. Powder Diffr..

[46] Finko M., Koroglu B., Rodriguez K. E., Rose T. P., Crowhurst J. C., Curreli D., Radousky H. B., Knight K. B. (2023). Stochastic Optimization
of a Uranium Oxide Reaction Mechanism Using Plasma Flow Reactor Measurements. Sci. Rep..

[47] Akiyama D., Akiyama H., Uehara A., Kirishima A., Sato N. (2019). Phase Analysis of Uranium Oxides after Reaction with Stainless Steel
Components and ZrO2 at High Temperature by XRD, XAFS, and SEM/EDX. J. Nucl. Mater..

[48] Wriedt H. A. (1991). The Fe-O
(Iron-Oxygen) System. J. Phase Equilib..

[49] Landa A., Söderlind P., Roehling J., McKeown J. T. (2025). Thermodynamics of
Liquid Uranium from Atomistic and Ab Initio Modeling. Appl. Sci..

[50] Gueneau C., Baichi M., Labroche D., Chatillon C., Sundman B. (2002). Thermodynamic Assessment of the Uranium – Oxygen
System. J. Nucl. Mater..

[51] Fink J. K. (2000). Thermophysical
Properties of Uranium Dioxide. J. Nucl. Mater..

[52] Cordfunke E. H. P. (1961). α-UO3:
ITS PREPARATION AND THERMAL STABILITY. J. Inorg.
Nucl. Clem..

[53] Zhang Y., Evans J. R. G., Yang S. (2011). Corrected Values for Boiling Points
and Enthalpies of Vaporization of Elements in Handbooks. J. Chem. Eng. Data.

[54] Sterrett K. F., Klement W., Kennedy G. C. (1965). Effect
of Pressure on the Melting
of Iron. J. Geophys. Res..

[55] Dobrosavljevic V. V., Zhang D., Sturhahn W., Chariton S., Prakapenka V. B., Zhao J., Toellner T. S., Pardo O. S., Jackson J. M. (2023). Melting
and Defect Transitions in FeO up to Pressures of Earth’s Core-Mantle
Boundary. Nat. Commun..

[56] Wang H., Liu B., Yang G., You C. (2023). Multistep Kinetic Study of Fe2O3
Reduction by H2 Based on Isothermal Thermogravimetric Analysis Data
Deconvolution. Int. J. Hydrogen Energy.

[57] Kulpa-Koterwa A., Ossowski T., Niedziałkowski P. (2021). Functionalized Fe3O4
Nanoparticles as Glassy Carbon Electrode Modifiers for Heavy Metal
Ions Detection—A Mini Review. Materials.

[58] Petrov Y.B., Udalov Y.P., Subrt J., Bakardjieva S., Sazavsky P., Kiselova M., Selucky P., Bezdicka P. (2009). Phase Equilibria
during Crystallization of Melts in the Uranium Oxide-Iron Oxide System
in Air. Glass Phys. Chem..

[59] Okamoto H. (2007). O-U (Oxygen-Uranium). J. Phase Equilibria Diffus..

